# Magnet ingestion in growing children: a multi-center observational study on single and multiple magnet incidents

**DOI:** 10.1038/s41598-024-55127-0

**Published:** 2024-02-25

**Authors:** Amani N. Alansari, Temur Baykuziyev, Tutku Soyer, Servet Melike Akıncı, Khalid Khalfan Al Ali, Adel Aljneibi, Nafea Hussain Alyasi, Muhammad Afzal, Amine Ksia

**Affiliations:** 1 Department of Pediatric Surgery, Hamad General Hospital, Doha, Qatar; 2 Department of Anesthesiology, ICU and Perioperative Medicine, Hamad General Hospital, Doha, Qatar; 3 Department of Pediatric Surgery, Faculty of Medicine, Hacettepe University, Ankara, Turkey; 4 Department of Pediatric Surgery, Al Qassimi Women and Children’s Hospital, Sharjah, United Arab Emirates; 5 Department of Pediatric Surgery, Sheikh Khalifa Medical City, Abu Dhabi, United Arab Emirates; 6 Department of Pediatric Gastroenterology, Sheikh Khalifa Medical City, Abu Dhabi, United Arab Emirates; 7 Department of Pediatric Surgery, Maternity and Children Hospital, Dammam, Kingdom of Saudi Arabia; 8 Department of Pediatric Surgery, Faculty of Medicine, Fattouma Bourguiba Hospital, Monastir, Tunisia

**Keywords:** Multiple magnetic foreign bodies, Magnet ingestion, Children, Surgery, Health care, Medical research, Signs and symptoms

## Abstract

Over the past 15 years, there has been a noticeable uptick in incidents involving children ingesting multiple magnetic foreign bodies which can cause injuries and gastrointestinal complications including death. The current study aimed to identify the prevalence, clinical presentation, and management of single or multiple magnet ingestions. A retrospective multi-central cross-sectional study was conducted to include all pediatric patients < 18 years presented to the emergency department with ingestion of single or multiple magnets and admitted across hospitals in Qatar, UAE, KSA, Tunisia, and Turkey between January 2011 and December 2021. Demographics, symptoms, management, and outcomes were analyzed. There were 189 magnet ingestions, of which 88 (46.6%) were multiple magnet ingestions. Most patients (55.6%) were male, and the median age was 3.9 (IQR 2–7) years. An abdominal X-ray was obtained in all cases. 119 (62%) patients were conservatively treated, 53 (28%) required surgical intervention and 17 (8.9%) underwent gastroscopy. None of the patients with single magnet ingestions experienced morbidity or severe outcomes. Multiple magnet ingestions led to significant morbidity including hospitalizations, perforations (44.3%), severe intestinal necrosis (19.3%), peritonitis (13.6%), severe abdominal infection (10.2%), and septic shock (4.5%). The rate of surgical intervention (59.1% vs. 1.0%) and gastroscopy (15.9% vs. 3.0%) was significantly higher in the multiple ingestion group compared to the single magnet ingestion group. No deaths were identified. A high risk of serious complications, including the need for surgery to remove the magnets and substantial morbidity may result from swallowing more than one magnet. Magnet safety requirements, public education, and improved legislation are urgently required.

## Introduction

Recently, there has been a significant rise in the incidence of magnet ingestion by children and this has been recognized as a growing health issue worldwide^1^, necessitating focused consideration^2,3^. Magnet ingestion-related morbidities, in general, are attributed to neodymium magnets, which are significantly smaller than older generation magnets (5 mm) and ten to twenty times more powerful. Neodymium magnets are available in hundreds of different forms, either as magnets themselves or as components of children's toys^4^. Neodymium magnets of 5 mm in size, can exert a force of up to a half kilogram between one another^5^. Ingestion of such strong multiple magnets can easily locate each other in the gastrointestinal tract within 12–48 h and can cause ischemia, necrosis, and perforation of intervening gut walls^6–8^. However, the initial phase of magnet ingestion is asymptomatic followed by non-specific symptoms such as pain, vomiting, or fever. Rarely do patients present with shock due to delayed reporting of magnets ingestion^9^. Thus, symptom origins remain elusive^8^. This means delineating the characteristics of cases of magnet ingestion is paramount, where commercial magnet applications are prevalent.

Magnet foreign body ingestion is a drastic health problem that has a particularly adverse impact on the health status of infants and young children. Especially between the ages of 6 months and 4 years^10^ by inadvertent swallowing of small objects such as small button batteries, magnetic beads, and other metallic items^3,11^. The detrimental outcomes of accidental magnet ingestion by this young age group cannot be overstated and necessitate immediate attention. The implications of this issue are numerous, ranging from discomfort and pain to serious consequences including surgical intervention and, in rare cases, death^5,7,12^. Therefore, healthcare professionals, parents, and caregivers must remain vigilant in their efforts to prevent such incidents^13^. Every effort should be taken to help mitigate the risk of harm and ensure the safety of the most vulnerable populations.

The accumulating evidence from the literature emphasizes the serious outcome of magnet foreign body ingestion on children's health^3,4,14^. Between 1995 and 2010, the Centers for Disease Control and Prevention (CDC) documented the fatalities of fourteen children under the age of thirteen as a result of battery ingestion^15^, Moreover, 481 cases of magnet ingestions were reported in the North American Society for Pediatric Gastroenterology, Hepatology, and Nutrition 2012 survey^16^. In addition to becoming lodged in the esophagus or other parts of the gastrointestinal tract, high-powered neodymium magnet beads can attract each other through the multilayer small intestine, which can result in intestinal obstruction, tissue necrosis, volvulus, fistulas, and intestinal perforation, and which can then lead to peritonitis^17,18^. Therefore, proactive measures should be taken, such as closely monitoring young children and ensuring that small objects that can be swallowed are kept out of their reach^19^.

Furthermore, multiple magnetic foreign body ingestion may need surgical intervention if esophageal-gastric-duodenal endoscopy (OGDE) either fails to extract the magnetic beads or they are inaccessible^20^. A multi-center analysis conducted by major trauma centers in the United Kingdom found that 51% of children needed surgical intervention despite early identification, diagnosis, and therapy^7,21^. Of these children, 89% received a laparotomy, and 19% needed an intestinal resection^21^. In a Japanese study, ODGE was successful in only 10–20% of children admitted with foreign body ingestions^4^. The decision of the surgical intervention and the type of the intervention is determined based on the multiplicity and the accessibility of the magnets as well as the presence of perforation and peritonitis^13,19,22,23^.

Therefore, a nuanced characterization of the features of children's ingestion of multiple magnetic foreign bodies is necessary to delineate the impact of this condition on the health status of children. However, no independent research has studied the differences and similarities between the ingestion of a single magnet as compared to multiple magnets. Therefore, the current study focused on investigating the demographic, clinical, and outcome feature differences between single and multiple magnet ingestion. Multi-center experiences from Qatar, the UAE, the KSA, Tunisia, and Turkey are represented in the current research. The results of this study help raise awareness of the gravity of multiple magnet ingestion among health practitioners for early detection, diagnosis, and proper management of the magnet ingestion dilemma. Additionally, the evidence from this study would encourage health authorities to take into consideration the necessary administrative and legislative measures to set the safety precautions to minimize the accessibility of children to magnets in the hope of reducing accidental ingestions of potentially serious foreign bodies.

## Materials and methods

### Study design

This is a retrospective cross-sectional study that included all magnet/magnets ingestion cases in children under 18 years of age. Those cases with incomplete records were excluded from the study. The eligible cases were admitted and treated at hospitals across Qatar, the UAE, the KSA, Tunisia, and Turkey between January 2011 and December 2021. Data was retrieved from the pediatric surgery database at Hamad General Hospital (Qatar), Maternity and Children Hospital (KSA), Al Qassimi Women and Children Hospital (UAE), Sheikh Khalifa Medical City (UAE), Hacettepe University (Turkey), and Fattouma Bourguiba University Hospital (Tunisia).

### Data collection

The data consists of demographic characteristics, clinical presentations (ranging from asymptomatic cases to those with gastrointestinal symptoms such as vomiting, fever, or abdominal pain), instances of witnessed ingestion, the duration of ingestion in hours, the type and number of magnets ingested (including neodymium balls, Rozets, discs, ellipses, bars, rectangular and ovoid stress magnets), and anatomical location of ingestion (hypopharynx, esophagus, stomach, small intestine, and large intestine). In addition, radiological investigations, and management approaches (ranging from conservative treatments to surgical interventions and gastroscopies) were obtained. Moreover, information was collected regarding the duration of effective conservative treatments in days, by emergency surgery, the number of magnet ingestions, lengths of stay (LOS) in the Intensive Care Unit (ICU) and hospital, complications (perforation, septic shock, peritonitis, severe abdominal infection, including purulent ascites or significant intestinal content leakage, cases of severe intestinal necrosis necessitating intestinal resection and anastomosis), as well as overall mortality.

### Ethical approval

Ethical approval was received from the Institutional Review Board (IRB) at Hamad Medical Corporation (MRC-01-22-460) in Doha, Qatar. Data were collected without any direct interactions with the individuals involved. This research has been performed per the Declaration of Helsinki and all methods were carried out according to relevant guidelines and regulations of MRC/HMC.

### Statistical analysis

Data analyses were performed using the Statistical Package for Social Sciences version 23 (SPSS Inc., Chicago, IL) and a Microsoft Excel spreadsheet was used to collect, tabulate, and analyze the data. Numerical data were presented in mean ± standard deviation (SD) or median and interquartile range (IQR) and the categorical data were presented as numbers and percentages (%). The chi-square and Fisher’s exact test were used to examine variations in categorical variables between groups. Continuous variables were assessed through the Students-t test and one-way ANOVA for parametric data. For non-parametric data, the Kruskal–Wallis test was used. Multivariable regression analysis was performed to determine the predictors of in-hospital complications using the most relevant factors such as age, sex, abdominal pain, duration of ingestion, number of magnets, surgical intervention, and gastroscopy. Data were expressed by the odds ratio (OR) and 95% confidence intervals (CIs). A significance level of less than 0.05 (p < 0.05) determined statistical significance.

## Results

### Descriptive statistics

A total of 189 pediatric patients with magnetic foreign bodies ingested were included in the study over the course of the 10-year study period, 55% male and 44% female. The median age of the patients was 3.9 (IQR 2–7) years, more than 50% were in the age range 0–4 years followed by the age group > 4 to 9 years (34.9%) and > 9 to ≤ 18 years (7.4%). The majority were asymptomatic (67%) and the remaining 33% presented with gastrointestinal symptoms (Table 1). Clinical presentations by descending order were abdominal pain (26.5%), vomiting (22.8%), and fever (9.5%). The median duration of ingestion was 4 (range 1–2160) hours and 60% of the events were witnessed by the family members. The most common types of magnets ingested were neodymium ball (75.7%) and Rozet (19%) with a median number of 1 (1–42) magnets ingested. The small intestine (51.9%) was the most common anatomical location of ingested magnets followed by the stomach (32.8%) and large intestine (24.3%).

**Table 1:** Characteristics of the pediatric patients with magnetic foreign body ingestion (total number = 189).

Age (years) median, IQR	3.9 (2–7)
0 to 4 years	109 (57.7%)
> 4 to 9 years	66 (34.9%)
> 9 to < 14 years	14 (7.4%)
Gender	
Male	105 (55.6%)
Female	84 (44.4%)
Gastrointestinal symptoms	
Abdominal pain	50 (26.5%)
Vomiting	43 (22.8%)
Fever	18 (9.5%)
Clinical presentation	
Asymptomatic	127 (67.2%)
Symptomatic	62 (32.8%)
Duration of ingestion (hours)	4 (1–2160)
Event witnessed	113 (59.8%)
Event unwitnessed	76 (40.2%)
Type of magnet	
Neodymium ball	143 (75.7%)
Rozet	36 (19.0%)
Ovoid stress magnet	5 (2.6%)
Rectangular	5 (2.6%)
Number of magnets (median, range)	1 (1–42)
Single	101 (53.4%)
Multiple	88 (46.6%)
Anatomical location	
Small intestine	98 (51.9%)
Stomach	62 (32.8%)
Large intestine	46 (24.3%)
Esophagus	6 (3.2%)
Broncos	1 (0.5%)

### Conservative versus surgical intervention and gastroscopy

Comparative analyses were performed by classifying cases based on management approaches into conservative, surgical intervention, and gastroscopy categories. All patients underwent abdominal radiography and only 2.6% had abdominal CT scanning. Sixty- three percent (63%) of the patients were managed conservatively; 28% required surgical intervention, while 9% underwent endoscopy (Table 2). In six cases initial conservative management was unsuccessful and these cases required surgical intervention.

**Table 2:** Management and outcome following ingestion of single and multiple magnetic foreign bodies.

Laboratory findings	
WBC (n = 79)	11.1 ± 4.5
Hemoglobin (n = 80)	11.9 ± 1.3
C-Reactive protein (n = 26)	12.8 (0.3–289)
X-ray abdomen	189 (100%)
Abdominal CT scan	5 (2.6%)
Management	
Conservative	119 (63.0%)
Surgical intervention	53 (28.0%)
Gastroscopy	17 (9.0%)
Conservative treatment duration (days) (n = 67)	2 (1–7)
Emergency surgery upon admission	39 (20.6%)
Number of attempts (n = 47)	1 (1–1)
ICU admission	11 (5.8%)
ICU LOS (days)	5 (1–13)
Hospital LOS (days)	1 (1–19)
Morbidity	81 (42.9%)
Perforation/fistula	39 (20.6%)
Severe intestinal necrosis**	17 (9.0%)
Peritonitis	12 (6.3%)
Severe abdominal infection*	9 (4.8%)
Septic shock	4 (2.1%)
Mortality	0 (0.0%)

*Purulent ascites or massive leakage of intestinal contents; **necessitating intestinal resection and anastomosis; LOS: length of stay; 6 cases initially treated conservatively failed and required surgery, 5 cases with conservative treatment had gastroscopy, and 3 who had gastroscopy also had surgical intervention.

**Figure 1:**
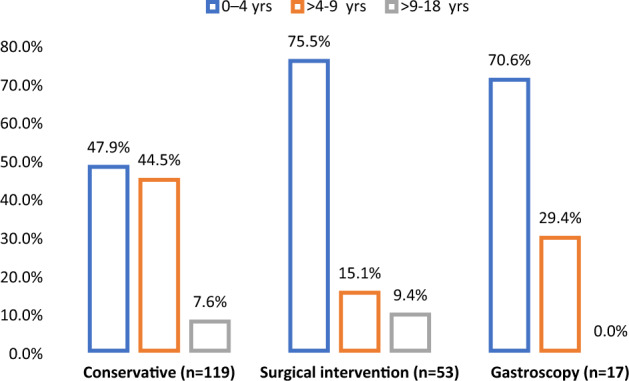
Management of magnetic ingestions by age groups.

Five cases with conservative treatment had gastroscopy, and 3 patients who had gastroscopy also had surgical intervention. Emergency surgery upon admission was indicated in 21% of cases and 5.8% of cases required ICU admission. The frequency of surgical intervention (75.5%) and endoscopy (70.6% in children within 4 years of age as compared to other age groups) is described (Fig. 1). The median hospital LOS was 1 (1–19) days. In-hospital complications included perforation (20.6%), severe intestinal necrosis (9%), and peritonitis (6.3%).

Patients treated conservatively were more likely to be older in age (P = 0.001), were asymptomatic on initial presentation (P = 0.001) and the majority ingested Rozet type of magnets (P = 0.001) as compared to other groups (Table 3). Sex distribution showed more male victims (P = 0.005) and magnets were predominantly located in the stomach (P = 0.008). As compared to other groups, those who require surgical intervention were more likely to be females (P = 0.005), frequently presented with abdominal discomfort (P = 0.001), vomiting (P = 0.001), and fever (P = 0.001), with a higher median duration of magnet ingestion (P = 0.001) and number of magnets (P = 0.001). In addition, the surgical group frequently ingested neodymium balls (P = 0.001), which were mainly identified in the small intestine (P = 0.001), and such patients were more likely to be admitted to the ICU (P = 0.001) and had a prolonged hospital stay (P = 0.001). Furthermore, the rate of in-hospital complications such as perforation (P = 0.001), septic shock (P = 0.03), peritonitis (P = 0.001), severe abdominal infection (P = 0.001), and severe intestinal necrosis (P = 0.001) was significantly higher in the surgical group, as compared to other groups.

**Table 3:** Clinical parameters and outcome by management.

	Conservative (n = 119)	Surgical intervention (n = 53)	Gastroscopy (n = 17)	P-value
Age (years) median, IQR	4.5 (3–7)	2.0 (1.6–4.1)	2.4 (1.8–4.5)	0.001
Gender				
Male	67 (56.3%)	23 (43.4%)	15 (88.2%)	0.005
Female	52 (43.7%)	30 (56.6%)	2 (11.8%)	
GI symptoms				
Abdominal pain	12 (10.1%)	35 (66.0%)	3 (17.6%)	0.001
Vomiting	4 (3.4%)	37 (69.8%)	2 (11.8%)	0.001
Fever	1 (0.8%)	17 (32.1%)	0 (0.0%)	0.001
Asymptomatic	104 (87.4%)	10 (18.9%)	13 (76.5%)	0.001
Duration of ingestion (hrs)	2 (1–168)	72 (1–2160)	14.5 (1–960)	0.001
Type of magnet				
Neodymium ball	77 (64.7%)	52 (98.1%)	14 (82.4%)	0.001
Rozet	32 (26.9%)	1 (1.9%)	3 (17.6%)	
Ovoid stress magnet	5 (4.2%)	0 (0.0%)	0 (0.0%)	
Rectangular	5 (4.2%)	0 (0.0%)	0 (0.0%)	
Number of magnets	1 (1–7)	6 (1–42)	4 (1–40)	0.001
Single magnet	97 (81.5%)	1 (1.9%)	3 (17.6%)	0.001
Multiple magnets	22 (18.5%)	52 (98.1%)	14 (82.4%)	
Anatomical location				
Small intestine	49 (41.2%)	45 (84.9%)	4 (23.5%)	0.001
Stomach	38 (31.9%)	13 (24.5%)	11 (64.7%)	0.008
Large intestine	28 (23.5%)	17 (32.1%)	1 (5.9%)	0.16
Broncos	0 (0.0%)	0 (0.0%)	1 (5.9%)	0.35
Esophagus	2 (1.7%)	0 (0.0%)	4 (23.5%)	0.001
ICU admission	0 (0.0%)	11 (20.8%)	0 (0.0%)	0.001
ICU LOS (days)	–	5 (1–13)	–	–
Hospital LOS (days)	1 (1–4)	7 (4–19)	1 (1–4)	0.001
Morbidity				
Perforation	0 (0.0%)	38 (71.7%)	1 (5.9%)	0.001
Septic shock	0 (0.0%)	4 (7.5%)	0 (0.0%)	0.03
Peritonitis	0 (0.0%)	12 (22.6%)	0 (0.0%)	0.001
Severe abdominal infection	0 (0.0%)	8 (15.1%)	1 (5.9%)	0.001
Severe intestinal necrosis	0 (0.0%)	17 (32.1%)	0 (0.0%)	0.001

### Single versus multiple magnet ingestion

Notably, the single magnet group had a significantly higher median age (4.3 vs. 2.4 years) than the multiple magnets group (P = 0.001; Table 4). Gastrointestinal symptoms were significantly more prevalent in the multiple magnets group, which included abdominal pain, vomiting, and fever (P = 0.001). Conversely, the single magnet group displayed a higher likelihood of being asymptomatic, whereas the multiple magnets group had a higher proportion of symptomatic cases (P = 0.001). In terms of ingestion duration, the multiple magnet groups had a significantly longer median duration (36 h) compared to the single magnet group (2 h), (P = 0.001). The type of magnet ingested also exhibited significant differences with Neodymium balls being more common in single magnet cases and Rozet magnets being predominant in the multiple magnets group.

**Table 4:** Comparison based on number of magnets ingested.

Variables	Single magnet (n = 101)	Multiple magnets (n = 88)	P-value
Age (years) median, IQR	4.3 (2.9–7.0)	2.4 (1.6–5.0)	0.001
Gender			
Male	60 (59.4%)	45 (51.1%)	0.25
Female	41 (40.6%)	43 (48.9%)	
Gastrointestinal symptoms			
Abdominal pain	8 (7.9%)	42 (47.7%)	0.001
Vomiting	2 (2.0%)	41 (46.6%)	0.001
Fever	1 (1.0%)	17 (19.3%)	0.001
Clinical presentation			
Asymptomatic	92 (91.1%)	35 (39.8%)	0.001
Symptomatic	9 (8.9%)	53 (60.2%)	
Duration of ingestion (h)	2 (1–168)	36 (1–2160)	0.001
Type of magnet			
Neodymium ball	62 (61.4%)	81 (92.0%)	0.001
Rozet	29 (28.7%)	7 (8.0%)	
Ovoid stress magnet	5 (5.0%)	0 (0.0%)	
Rectangular	5 (5.0%)	0 (0.0%)	
Anatomical location			
Small intestine	37 (36.6%)	61 (69.3%)	0.001
Stomach	38 (37.6%)	24 (27.3%)	0.13
Large intestine	21 (20.8%)	25 (28.4%)	0.22
Bronchus	0 (0.0%)	1 (1.1%)	0.28
Esophagus	2 (2.0%)	4 (4.5%)	0.31
Management			
Conservative	97 (96.0%)	22 (25.0%)	0.001
Surgical intervention	1 (1.0%)	52 (59.1%)	
Gastroscopy	3 (3.0%)	14 (15.9%)	
ICU admission	0 (0.0%)	11 (12.5%)	0.001
ICU LOS (days)	–	5 (1–13)	–
Hospital LOS (days)	1 (1–6)	5 (1–19)	0.001
Morbidity			
Perforation	0 (0.0%)	39 (44.3%)	0.001
Septic shock	0 (0.0%)	4 (4.5%)	0.03
Peritonitis	0 (0.0%)	12 (13.6%)	0.001
Severe abdominal infection	0 (0.0%)	9 (10.2%)	0.001
Severe intestinal necrosis	0 (0.0%)	17 (19.3%)	0.001

*IQR* interquartile range, *ICU* intensive care unit, *LOS* length of hospital stay.

The anatomical location of magnet ingestion differed significantly, with the multiple magnets group more likely to involve the small intestine. Management strategies differed, as the single magnet group was more often treated conservatively, while the multiple magnets group required more surgical intervention and endoscopy (P = 0.001). Notably, the multiple magnets group had longer ICU and hospital LOS compared to the single magnet group (P = 0.001). In terms of morbidity, the multiple magnets group experienced significantly higher rates of complications, including perforation, septic shock, peritonitis, severe abdominal infection, and severe intestinal necrosis.

### In-hospital complications

Predictors for in-hospital complications were estimated using multiple regression analysis. After adjusting for the relevant confounding factors, such as age, gender, duration of magnet ingestion, and number of magnets, only abdominal pain (OR 21.988, 95% CI 1.342–360.503; p = 0.03) and surgical intervention (OR 163.4, 95% CI 11.30–2361.57; p = 0.001) were found to be the independent predictors of in-hospital complications (Table 5).

**Table 5:** Multivariate regression analysis for the predictors of in-hospital complications.

Variables	Odd ratio	95% Confidence interval	P-value
Age (years)	0.661	0.459–0.952	0.026
Male gender	3.406	0.210–55.138	0.388
Abdominal pain	21.998	1.342–360.503	0.030
Duration of ingestion	1.002	0.998–1.006	0.410
Number magnets	1.127	0.971–1.307	0.115
Surgical intervention	163.4	11.30–2361.57	0.001
Gastroscopy	0.143	0.004–5.031	0.285

## Discussion

Ingestion of multiple foreign magnet bodies has raised alarm due to the increased rates of hospital admissions with serious complications, as compared to single magnet ingestions. An earlier study showed that 15.7% of patients were admitted due to multiple magnet ingestions versus 2.3% of single magnet ingestions^24^. A recent study found that 31% of cases had multiple magnet ingestions with 36% of these requiring endoscopies^25^. Another study reported that nearly half of the cases had single magnets while the other half showed either 2 or more than 3 magnet ingestions^8^. Accumulating evidence from the literature emphasized that multiple magnet ingestion is more serious and increases the risk of hospitalization, surgical intervention, and complications^8,26,27^.

Magnet foreign body ingestion is most common in children under the age of 4^19^ between the ages of 6 months and 4 years old^10^. According to the studies conducted by the American Association of Poison Control Centers, 75% of the reported ingestions were in children who were five years old or younger^16^. Moreover, a cross-sectional study conducted in the US from 2002 to 2010 reported that more than 22,000 cases of multiple magnet ingestions in children were observed with a median age of 4.7 years^11^. The current study concurred with the literature that most children were under the age of 4. This behavior can be attributed to children's natural curiosity and explorative nature. As children start moving and exploring their surroundings, they tend to test everything they come across by putting it in their mouths. Small and shiny objects like magnets can easily attract a child's attention. This means they can be easily swallowed and associated with complications. Therefore, parents and caregivers need to ensure that young children are not exposed to small magnets and other potentially hazardous objects^13^.

In the current study, single magnet ingestion usually occurs in older children compared to multiple magnet ingestion which is more common among younger children with no significant sex difference. The median duration of multiple magnet ingestion extends to 36 h and only 2 h in single magnet ingestion. Children who ingested single magnets were mostly asymptomatic while those who ingested multiple magnets presented gastrointestinal symptoms including abdominal pain, vomiting, and fever. Neodymium balls are the most common magnets type ingested by children followed by Rozets. Moreover, the study revealed that the small intestine is the most significantly affected organ in multiple as compared to single magnet ingestions. Other organs such as the stomach, large intestine, bronchus, and esophagus were the seat of magnet foreign body impacts with no significant differences between both groups. It was obvious from the results of this study that children with multiple magnet ingestions are prone to surgical interventions and endoscopies. Children with single magnet ingestions were usually managed conservatively. In addition, multiple magnet ingestion increases the risk of ICU admission, and LOS as well as serious complications including intestinal perforation, septic shock, peritonitis, severe abdominal infection, and severe intestinal necrosis. As a result of the regression analysis, in-hospital complications are more likely to occur due to abdominal pain and surgery independent of other factors such as age, sex, duration of magnet ingestion, or the number of ingested magnets. No mortality was reported in this study.

In the first few hours of magnet ingestion, the child is asymptomatic unless witnessed. Therefore, unwitnessed magnet ingestion was associated with prolonged duration from ingestion to hospital admission^3^. Most of the children observed in our study were cases of single magnet ingestion, witnessed and asymptomatic, thus the outcome was favorable.

All cases in this study were radiologically examined by plain X-ray (AP and lateral view) on the neck, chest, and abdomen because of the high diagnostic yield of plain X-ray^1,11^. CT scans were reserved for complicated cases and used to detect inflammation and small perforations; shown as localized pneumoperitoneum thick bowl segment^28^. Due to the magnetic nature of the ingested foreign body, MRI was contraindicated^6^.

**Figure 2:**
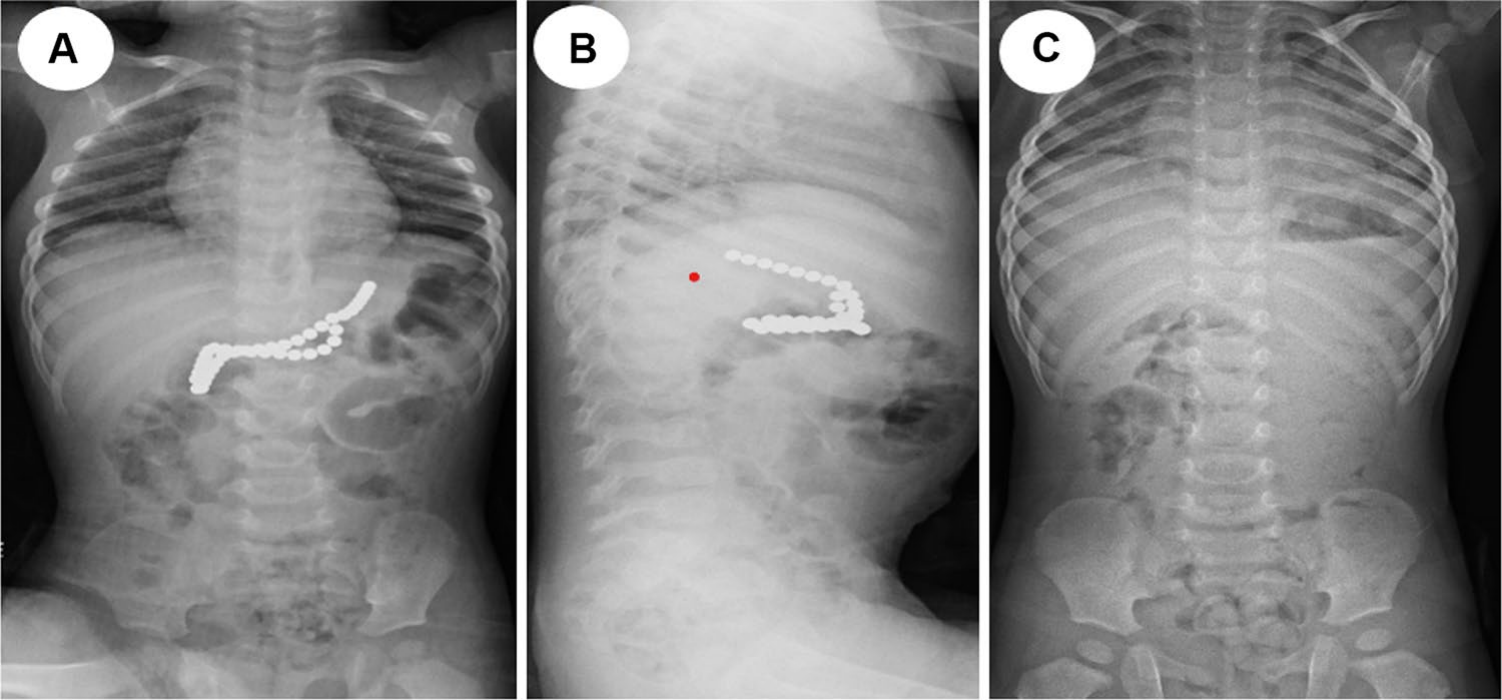
Radiograph showing 40 magnets removed endoscopically from stomach and duodenum. (**A**) AP view; (**B**) Lateral view; (**C**) after removal of magnets.

**Figure 3:**
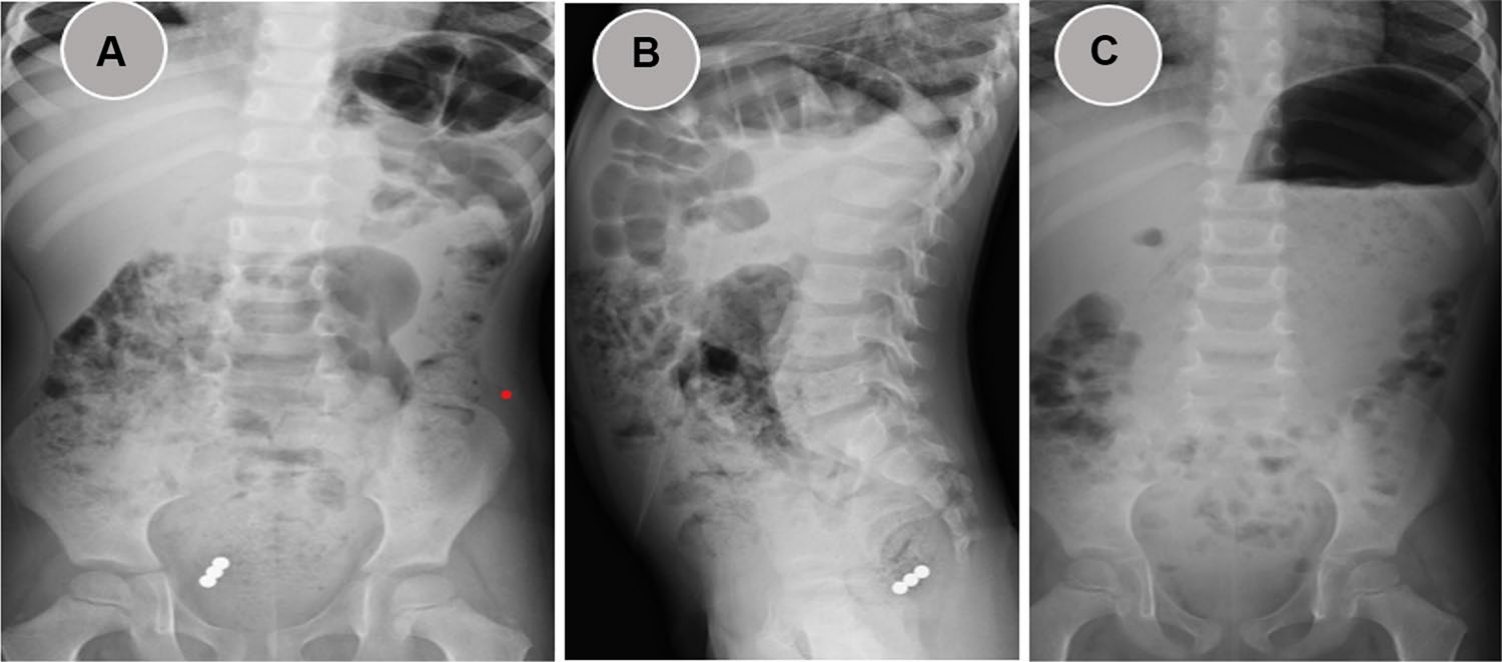
Radiograph showing 3 attached magnets without intervening tissue passed naturally (**A**) AP view; (**B**) Lateral view; (**C**) after evacuation of magnets.

**Figure 4:**
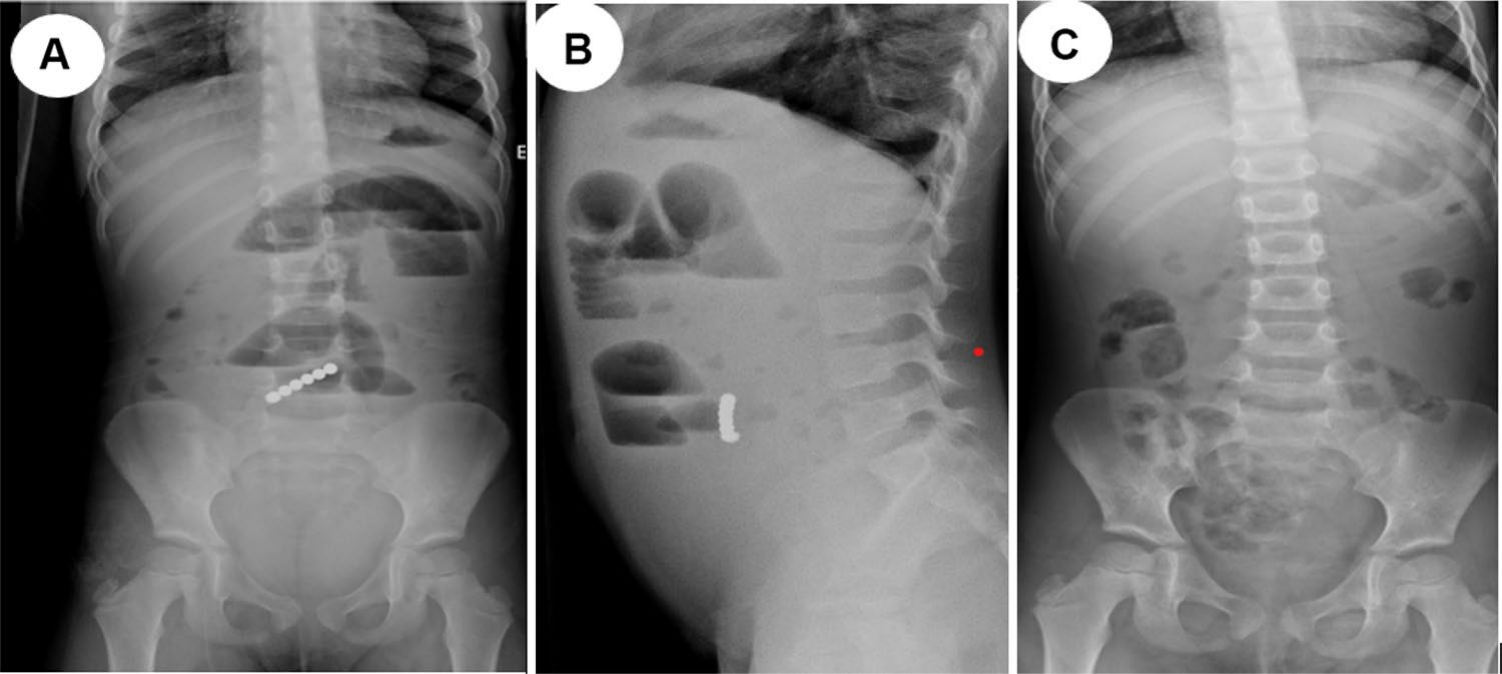
Preoperative radiograph showing multiple magnets causing intestinal obstruction (multiple air fluid levels) (**A**) AP view; (**B**) Lateral view; (**C**) after surgical removal of magnets.

**Figure 5:**
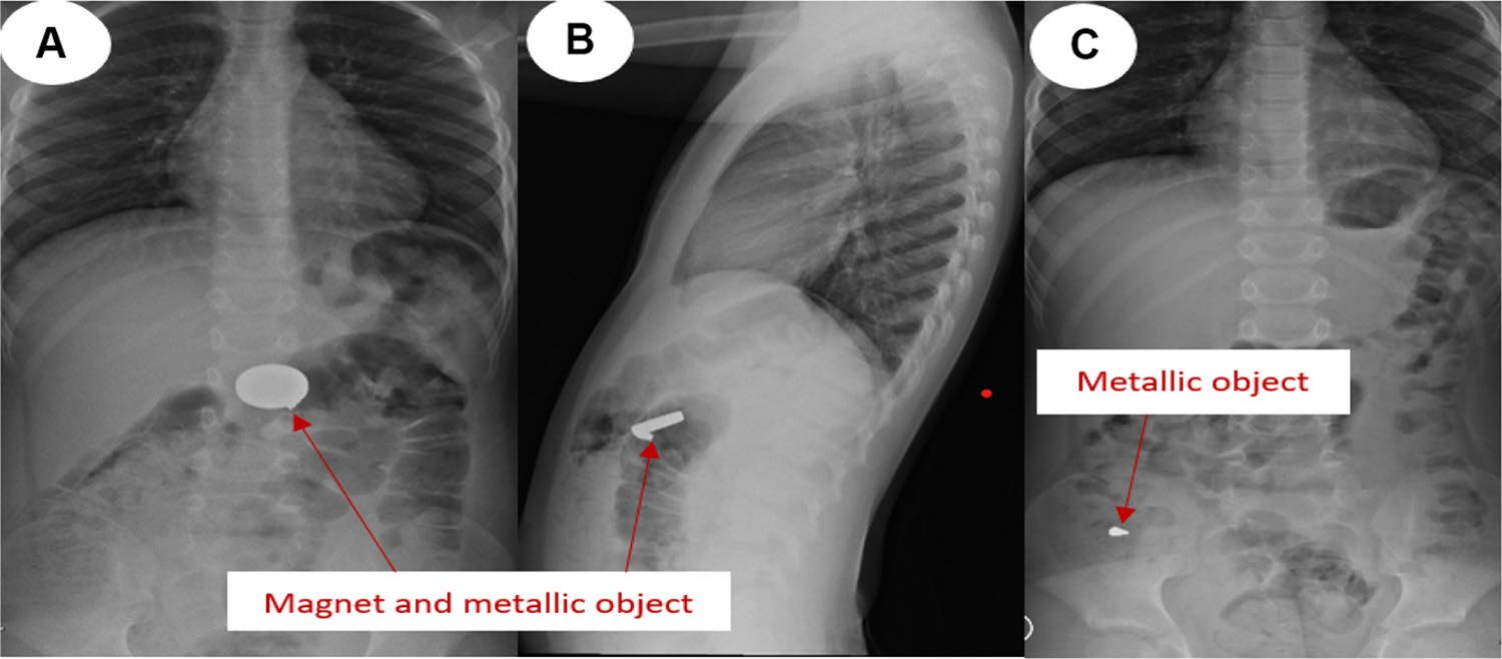
Radiograph showing magnets and metallic objects. Magnet extracted endoscopically and metallic object passed spontaneously.

**Figure 6:**
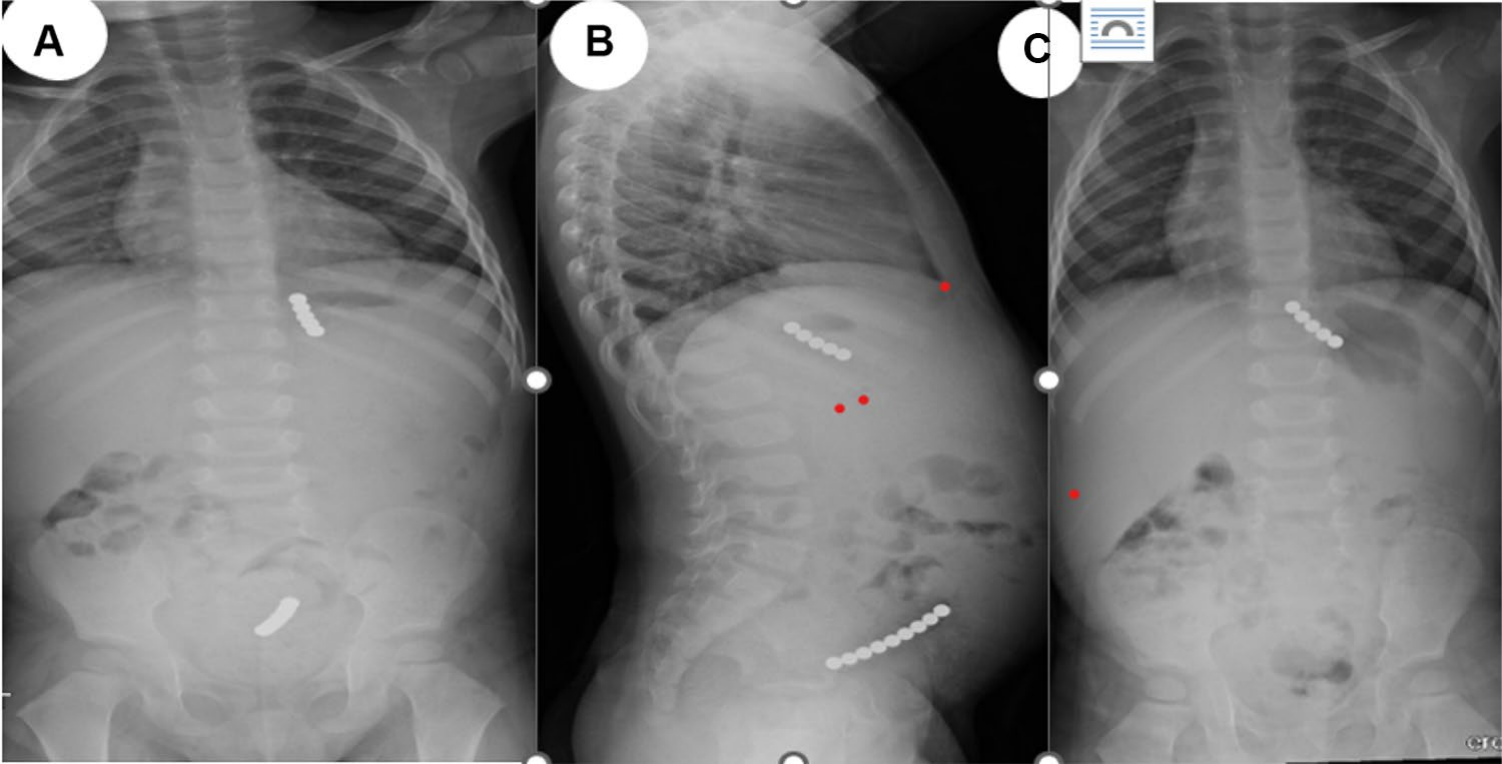
Radiograph showing multiple magnets (**A**) AP view; (**B**) Lateral view; (**C**) lower set of magnets passed after fleet enema and upper set removed endoscopically.

We provide a sample of situations from our research in which the management was tailored to the specific circumstances. All these instances demonstrate that to effectively handle a case, it is necessary to assess and personalize it on an individual basis. One patient presented with a history of witnessing ingestion of 40 magnets within 4 h of ingestion. All magnets were retrieved endoscopically from the stomach and duodenum (Fig. 2). In one asymptomatic patient, with witnessed ingestion, an abdominal radiograph showed 3 magnets matted together in the pelvis (meaning, these were progressing), an expectant option was adopted, and all passed uneventfully (Fig. 3). An emergency laparotomy was performed in one patient, because of intestinal obstruction caused by multiple magnet ingestions (Fig. 4). The abdominal radiograph of one asymptomatic patient who witnessed the ingestion of one-day duration showed a magnet attached to a metallic object in the upper abdomen. An endoscopy was performed, and a magnet was found at the pyloric area of the stomach and was removed while the metallic object was not visualized and it was left as such, which passed spontaneously later (Fig. 5). The abdominal radiograph of one mild symptomatic patient of unwitnessed ingestion, showed two sets of magnets, one at the upper central abdomen (assessed as magnets in the stomach) and one at the pelvic area (meaning, this set had moved lower down). A Fleet enema was administered, and a pelvic set of magnets passed per rectum while the upper set was removed endoscopically (Fig. 6).

Concerning interventions, most patients (49%) received either surgery, endoscopy, or both^27^. Another study showed that nearly 75% of children were administered either a laparotomy or laparoscopy for the management of foreign body magnet ingestion^29^. Similarly, this study showed that 94% of multiple magnet ingestion cases were managed either by surgery or endoscopy as compared to 81% of single magnet ingestion cases which were conservatively managed.

Although magnets can be found in any part of the gastrointestinal tract, the small intestine is the predominant site in cases of multiple ingestions compared to single ingestion cases^29^. Intestinal resection was carried out in 22% of children^4^. A case series reported small intestine affection in the jejunal and ileum^30^. A recent multi-center cross-sectional study reported that the initial site of multiple magnet ingestion was the stomach (52%) followed by the small intestine (29%)^31^. This study found that nearly 69% of multiple magnet ingestion cases had their small intestine afflicted. Although other parts of the gastrointestinal tract are also affected, this was to a lesser degree and had lower statistical significance as compared to single and multiple magnet ingestions. Postponing the pursuit of medical intervention allows magnetic beads to descend the gastrointestinal tract to ultimately arrive at the intestine.

Intestinal perforation was detected in 50% of cases with multiple magnet ingestions^30^. This is supported by a Chinese study that found gastrointestinal perforation to be the most common complication of multiple magnet ingestions (41.5%)^32,33^. Further, another study also found the small intestine to be the seat of magnet ingestion in 38% of cases^34^. Small intestinal fistulae and perforations were reported in a case series of multiple magnet ingestions^35^. No child with a single magnet ingestion suffered from intestinal complications.

Multiple magnet ingestion is associated with hospitalization, increased LOS, and on certain occasions ICU admission. One study showed that the LOS for 233 children with multiple magnet ingestion was 3 days and only 4 patients required ICU admission^27^. Another study showed that 75% of children required hospitalization for multiple magnet ingestions^14^. A multi-center study reported that the median hospital stay for magnet ingestions was 1.0 (0–3.0) days^31^. This study demonstrated that the LOS increased in patients who needed surgical intervention reaching 20.8% (n = 53) of the total patients who underwent surgery. In cases of multiple magnet ingestion, the hospital stay extended to a median of 5 (1–13) days compared to single magnet ingestions of 1 (1–6) days with significant statistical differences. Those with multiple ingestions required an estimated median of 5 (1–19) days in ICU. In contrast, no ICU admission occurred in single magnet ingestions.

### Strengths and limitations

The main strength of this study is the characterization of multiple as compared to single magnet ingestions. The study highlights the need to be aware of the risk of unintentional magnet intakes, and the need to obtain medical assistance and manage the situation promptly. The study provides evidence of the potential harm of multiple magnets to the health of children in terms of increasing risk of hospitalization, surgical interventions, and serious morbidities. Consequently, the study urges the profound need to restrict the access of children to potentially harmful magnetic beads and Rozets.

Although it is presumed that the data was recorded with scrutiny, there is a limitation due to the potential inherited risk of bias during the recording process. Moreover, the registered data is only for those who were presented to the emergency room and admitted to the hospital. Outpatients were not recorded.

## Conclusions

When a child ingests multiple magnets, the consequences can be severe and even life-threatening. Magnets can cause significant harm to the intestines including perforations, peritonitis, infections, and necrosis. When these complications arise, they require immediate surgical intervention and may necessitate prolonged hospital stays and/or ICU admissions.

It is important to understand that magnets have the potential to cause long-term health problems. Parents and caregivers should ensure that toys or objects with magnets are kept out of reach. It is essential to immediately seek medical attention if a child has ingested magnets or if there is any suspicion of this having occurred. Moreover, establishing robust Product Safety Regulations/legislations and standards for magnetic products in collaboration with relevant authorities is pivotal. By collectively implementing these measures, the region can reduce pediatric magnet ingestion cases and serve as a model for other regions.

## Data Availability

Data of this article will be available from the corresponding author upon reasonable request.

## Abbreviations

MRI: Magnetic resonance imaging
CT: Computed tomography scan
