# Author Correction: Magnet ingestion in growing children: a multi-center observational study on single and multiple magnet incidents

**DOI:** 10.1038/s41598-024-74550-x

**Published:** 2024-10-10

**Authors:** Amani N. Alansari, Temur Baykuziyev, Tutku Soyer, Servet Melike Akıncı, Khalid Khalfan Al Ali, Adel Aljneibi, Nafea Hussain Alyasi, Muhammad Afzal, Amine Ksia

**Affiliations:** 1 Department of Pediatric Surgery, Hamad General Hospital, Doha, Qatar; 2 Department of Anesthesiology, ICU and Perioperative Medicine, Hamad General Hospital, Doha, Qatar; 3 Department of Pediatric Surgery, Faculty of Medicine, Hacettepe University, Ankara, Turkey; 4 Department of Pediatric Surgery, Al Qassimi Women and Children’s Hospital, Sharjah, United Arab Emirates; 5 Department of Pediatric Surgery, Sheikh Khalifa Medical City, Abu Dhabi, United Arab Emirates; 6 Department of Pediatric Gastroenterology, Sheikh Khalifa Medical City, Abu Dhabi, United Arab Emirates; 7 Department of Pediatric Surgery, Maternity and Children Hospital, Dammam, Kingdom of Saudi Arabia; 8 Department of Pediatric Surgery, Faculty of Medicine, Fattouma Bourguiba Hospital, Monastir, Tunisia

Correction to: *Scientific Reports*10.1038/s41598-024-55127-0, published online 25 February 2024

The Acknowledgements section in the original version of this Article was incomplete.

The APC of this article was generously funded by the Qatar National Library (QNL).

now reads:

The APC of this article was generously funded by the Qatar National Library (QNL) and Hamad Medical Corporation’s Medical Research Center (MRC).

The original Article has been corrected.

